# Sirtuin mediated neuroprotection and its association with autophagy and apoptosis: Studies employing transgenic *C. elegans* model

**DOI:** 10.1186/1750-1326-8-S1-P65

**Published:** 2013-10-04

**Authors:** Aamir Nazir, Pooja Jadiya

**Affiliations:** 1 CSIR-Central Drug Research Institute, Lucknow, India

## Background

Sirtuins, NAD+-dependent protein deacetylases, are well known for their role in longevity. Aging is the major known risk factor for the onset of Parkinson’s disease (PD). In our previous studies, SIRT1 induction by calorie restriction (CR) diet regimen has been shown to protect against dopaminergic neurodegeneration via *sir-2.1* mediated pathway. Taking the studies forward, we endeavoured to understand the role of nicotinamide adenine dinucleotide activated protein deacetylase Sir2p/Sirt1 in calorie restriction mediated prevention of Parkinsonism employing transgenic *Caenorhabditis elegans* expressing human alpha synuclein.

## Materials and methods

We took advantage of the powerful genetics of model system *C. elegans* towards achieving our objectives. Transgenic *C. elegans* models - NL5901 (P_unc-54_::alphasynuclein::YFP+unc-119) expressing ‘human’ alpha synuclein; BZ555 (P*_dat-1_*::GFP) expressing green fluorescence protein (GFP) specifically in eight dopaminergic (DA) neurons and DA2123 expressing LGG-1::GFP, were employed for the studies. Gene silencing was achieved by employing RNAi methodology and the role of sirtuin was assayed by studying various parameters including alpha synuclein aggregation, content of reactive oxygen species, content of lipds, content of mitochondria and content of neurotransmitter dopamine.

## Results

Our findings provide evidences towards the role of calorie restriction in reducing α-synuclein aggregation, mitochondrial content, lipid content and ROS in human α-synuclein expressing strain of *C. elegans*. RNAi of *sir-2.1* enhanced aggregation of alpha synuclein but *sir-2.1* silenced worms raised on reduced calorie diet didn’t show protective effect in reducing protein aggregation which proved that protective effects of calorie restriction were mediated by NAD-dependent histone deacetylase activity of *sir-2.1*. We next focused on the mechanism of the *sir-2.1* mediated neuroprotective effect via autophagy. Assessment of autophagy in *C. elegans* was performed using a transgenic strain DA2123, expressing LGG-1::GFP. Worms with RNAi induced gene silencing of *sir-2.1* showed decreased expression of LGG-1::GFP and decreased mRNA level of different autophagy genes including *bec-1*,*atg-5*,*atg-7*, *lgg-1* and *atg-13* in qPCR studies. In order to further examine the signaling pathways regulating the SIRT1 mediated regulation of autophagic degradation, we analyzed the expression of different apoptosis genes including *ced-4*, *cep-1*, *lin-35*, *jkk-1* and *jnk-1*. In our studies, silencing of *sir-2.1* showed significant up-regulation of *ced-4* (Apaf-1) and *cep-1* (p53 ortholog- DNA damage pathway) apoptosis genes.

## Conclusions

Our study provides evidence for the protective role of *sir-2.1* on autophagosome formation in *C. elegans*, which is associated with the p53 and apaf-1 dependent signaling pathways; the well-known stress resistance mediators.

